# Shared Decision-Making Model for Adolescent Smoking Cessation: Pilot Cohort Study

**DOI:** 10.3390/ijerph182010970

**Published:** 2021-10-19

**Authors:** Kuan-Lun Chen, Yun-Chen Hsu, Yi-Hsuan Li, Fei-Ran Guo, Jaw-Shiun Tsai, Shao-Yi Cheng, Hsien-Liang Huang

**Affiliations:** 1 Department of Medicine, National Taiwan University, Taipei 10002, Taiwan; b06401014@ntu.edu.tw; 2 Department of Family Medicine, National Taiwan University Hospital, Taipei 10002, Taiwan; 106491@ntuh.gov.tw (Y.-C.H.); leemichelle74@gmail.com (Y.-H.L.); fjguo1@ntu.edu.tw (F.-R.G.); jawshiun@ntu.edu.tw (J.-S.T.); scheng2140@gmail.com (S.-Y.C.); 3 Department of Nursing, National Taiwan University Hospital, Taipei 10002, Taiwan

**Keywords:** adolescent smoking cessation, shared decision-making, tobacco control, early intervention

## Abstract

The control of tobacco use in adolescents is a critical public health issue that has long been studied, yet has received less attention than adult smoking cessation. Shared decision making (SDM) is a method that highlights a patient’s preference-based medical decision. This study aimed to investigate the effects of a novel SDM-integrated cessation model and early intervention on the control of tobacco use in adolescents. The SDM-integrated model provides psychological support and motivational enhancement by involving the participants in making decisions and plans through the three-talk model of the SDM principle. The primary outcome shows positive effects by both increasing the cessation rate (a 25% point abstinence rate at 3 month follow up) and decreasing the number of cigarettes smoked per day (60% of the participants at 3 month follow up) among 20 senior high school participants (mean age, 17.5 years; 95% male). The results also show that the model can achieve the goal of SDM and optimal informed decision making, based on the positive SURE test and the satisfaction survey regarding the cessation model. The SDM cessation model can be further applied to different fields of adolescent substance cessation, yielding beneficial effects regarding reducing potential health hazards. The dissemination of the model may help more adolescent smokers to cease smoking worldwide.

## 1. Introduction

Aside from the rapidly emerging use of e-cigarettes, which has gained a lot of attention during the past decade, adolescents’ use of cigarettes is remains a universal and long-lasting public health issue. According to a statistical report from the Taiwanese Health Promotion Administration, the smoking rates (referring to cigarette smoking unless specified in the rest of this article) of junior high school students and high school students were 3.0% and 8.4% in 2019, respectively. Additionally, 72% of adult smokers admitted their age of smoking initiation was under 18 in Taiwan [1]. According to WHO’s global report on trends in the prevalence of tobacco use, at least 43.8 million (12%) adolescents aged between 13 and 15 years used some form of tobacco over the past decade. For cigarette smoking, around 24 million (6.5%) adolescents aged between 13 and 15 years report that they currently smoke cigarettes. Although the estimated prevalence of tobacco use in late adolescents and young adults aged between 15 and 24 years declined since 2000, and the predicted prevalence is 14.2% in 2025, it is still challenging to maintain the descending trend [2]. Some studies also showed a doubled proportion of early adulthood initiation, and an increasing trend in daily smoking that has emerged in the past decade [3,4]. The early initiation of smoking is also related to health consequences such as decreased cardiovascular and pulmonary functions, multiple cancers, nicotine addiction, and psychological and behavioral issues due to a greater amount of exposure [5]. Additionally, according to recent studies, it is possible to prevent e-cigarette use and its potential harm, as current cigarette users have a higher rate of e-cigarette use [6,7]. Considering the adolescent smoker population and health consequences, it would be beneficial if the use of tobacco in adolescent smokers could be controlled at an early stage; further costs and health hazards caused by tobacco abuse or exposure would be reduced or eliminated.

Adolescents’ behaviors are characterized by significant positive and negative plasticity; their decisions are easily influenced by families, peers, their environment, media, and social media. A systematic review noted that family members have significant impacts on increasing the odds ratio of adolescent smoking by increasing the exposure, availability, and behavior surrounding smoking [8]. A systematic review and meta-analysis focusing on the relationship between adolescent peer pressure and smoking behavior revealed that peer pressure had a significant impact on increasing the prevalence of cigarette smoking among students (OR = 2.68), highlighting the importance of peers and the social environment [9]. A national study in Malaysia indicated that adolescents are more vulnerable to becoming smokers if they are exposed to tobacco promotions. In contrast, those who receive better anti-smoking education have a lower risk of smoking [10]. A systematic review also suggested that behavioral support and encouraging positive perceptions of cessation treatments improves cessation adherence and compliance [11]. To achieve the control of tobacco use in adolescents, a multidisciplinary plan providing enough cessation knowledge and environmental, behavioral, and social support is critically important. Although there are several statistical and mathematical models designed to assist decision-making analysis and increase decision consistency in multistep and multifactor decision-making situations, these models are difficult to apply clinically due to the above-mentioned complexity of adolescent smoking cessation [12].

Shared decision making (SDM) is defined as an approach in which clinicians and patients make decisions together using the best available evidence. This is an ideal interactive model that provides knowledge, support, and the exchange of opinions between clinicians and patients, highlighting patients’ autonomy [13]. During the SDM process, clinicians provide and explain available treatment options. Consensus between clinicians and patients is ideally reached after this discussion. With SDM, smokers can gain more knowledge about upcoming treatments, along with comparisons of adverse effects, and involve themselves in making the most suitable decision. Previously, shared decision making was not used often in adolescent smoking cessation; however, similar ideas can be found in previous studies. A study considering smoking cessation apps showed that it is important for the apps to take the user’s needs into consideration while responding to user-provided changes, which is similar to the concept of SDM [14]. Additionally, a study regarding patient decision aid published in 2006 proved that decision aid tools have positive effects on motivation, and on confidence in using and finishing cessation treatments. The study also suggested that decision aid tools increase smokers’ knowledge of smoking cessation and helps them to make better choices, while lessening room for excuses [15]. SDM has the potential to provide critical elements for adolescent smoking cessation; therefore, we aimed to integrate SDM into an adolescent tobacco control model.

The widely adopted transtheoretical model for smoking cessation proposes that five sequential stages of behavior change, namely, precontemplation, contemplation, preparation, action, and maintenance, can assist when designing strategies. Most adolescents are in the early three stages (the precontemplation, contemplation, and preparation stages). Intervention strategies should be customized and tailored to these stages [16]. The SDM model can be integrated and work jointly with the transtheoretical model, providing substantial help in staging and customizing. There are also some key differences between the SDM-integrated model and current adolescent tobacco control strategies. Current adolescent tobacco control strategies focus on two major principals: 1. preventing the initiation of cigarette smoking; 2. helping current smokers to quit [17]. The SDM-integrated method focuses on helping current smokers quit, perhaps achieving a “snowball” effect by reducing peer pressure and environmental exposure. As the use of pharmacotherapy still lacks sufficient clinical trial data and remains incongruent with several evidence-based guidelines, cessation counseling services and health education play major roles in adolescent tobacco cessation strategies [17]. Recent studies regarding adolescent tobacco cessation are rare; they mainly focused on e-cigarette cessation and the development of cessation mobile apps. These studies did not mention shared decision making; however, similar concepts and strategies could be taken into consideration. Two studies regarding adolescent smoking cessation using text message and smartphone apps showed a positive effect and a cessation rate of 31% using an interactive, supportive, self-motivating design, which are important features [18,19]. A recent systematic review highlighted the principles and effectiveness of multiple behavioral interventions and personalized counseling, which are also important in our SDM model [17]. In addition to the conventional, fundamental “offered” services, the SDM-integrated method ought to provide a more interactive, motivative, supportive, and preference-based cessation model by involving and collaborating with the smoker. In this study, we aimed to establish the SDM model for adolescent smoking cessation with the integration of motivational interviews and brief counseling. The model may be applied globally to assist more adolescent smokers in smoking cessation.

## 2. Materials and Methods

### 2.1. Study Design

The study was designed and conducted from March 2020 to March 2021 in a national medical center in Taiwan. The target participants were senior high school students from a senior high school close to the hospital, that had a health promotion project in collaboration with the hospital. Information regarding the adolescent smoking cessation SDM model was distributed using bulletin board posters and the school broadcasting system. Students who were adolescent smokers could join the model freely without having their assessments influenced by teachers. Participant recruitments were conducted under the participants’ own will, without any additional reward.

### 2.2. Framework of the SDM Model for Adolescent Smoking Cessation

The SDM model for adolescent smoking cessation was developed by integrating the SDM principle into the smoking cessation procedure (after a careful literature review by authors with clinical experience) via “team talks”, “option talks”, and “decision talks” [20,21].

**Figure 1:**
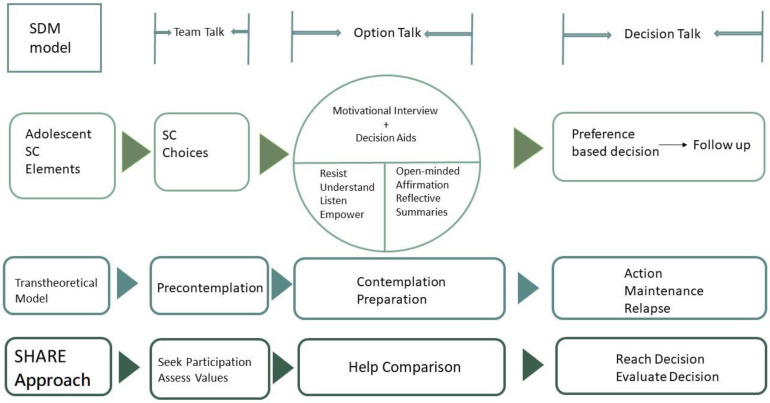
Framework of shared decision-making model for adolescent smoking cessation (SC).

The step “team talk” was initiated by smoking cessation educators (the authors Y.H Li and Y.Ch. Hsu) and focused on providing the adolescent smokers with possible smoking cessation choices. Furthermore, the motivations behind smoking cessation were explored. The understanding of adolescent smokers’ motivation is essential in the “team talk” and the “seek participation” component in the SHARE approach in SDM, proposed by the Agency for Healthcare Research and Quality (seek participation, help comparison, assess values, reach Decision, evaluate Decision) [20,21,22,23]. Additionally, according to the traditional smoking cessation theory of behavior change in the transtheoretical model, most students in this step were in the pre-contemplation or contemplation stage, as shown in Figure 1.

The goal of the “option talk” was for the educators to help the adolescents understand the risks and benefits of smoking cessation management when they entered the transtheoretical model of contemplation or preparation stages. In our model, this step consisted of a communication section with the educators, using the help of decision aid, such as a structured pamphlet. The pamphlet was a decision support tool with three parts: (1) an overview of the pros and cons of smoking cessation managements; (2) questions that help adolescent smokers to clarify their preferences toward the choices; and (3) the SDM quality assessment questionnaires, such as the SURE test. The GRADE (Grading of Recommendations Assessment, Development and Evaluation) and the requirement for patient decision aids in ethics, quality-of-care, and evidence-based medicine were used to develop the decision support tool [24,25]. An expert panel consisted of five physicians and two nurses who acted as smoking cessation educators and participated during the development phase. Moreover, the most important aspect of the design of our model during this step was trying to help adolescent smokers move from the preparation stage of the transtheoretical model to action. The concepts of motivational interviews and brief counseling were integrated in the communication between students and educators. Therefore, we incorporated two communication strategies into this step using the mnemonics “RULE” and “OARS” [26,27]. “RULE” consisted of four features of motivational interviews: “resist the righting reflex, understand your patient’s motivations, listen to your patient, empower your patient”. “OARS” represented four components of brief counseling: “open-ended questions, affirmation, reflective listening, summaries”. These strategies led the educators to incorporate humanistic communication into the “option talk” and build better rapport with the adolescent smokers.

The goal of the “decision talk” was to make decisions based on adolescents’ preferences after deliberation, and before entering the action stage of the transtheoretical model for smoking cessation, it was preferred if a concordant goal between adolescent smokers and educators had been reached.

### 2.3. Outcome Measurements and Analysis

The primary outcome that was assessed was three month abstinence of the adolescent smokers, and the change in the number of the cigarettes smoked per day after entering the model was also assessed one week, one month, and three months after entering the model. The above-mentioned data were participants’ self-reported data obtained from the questionnaire. If the participant indicated no cigarette use prior to the day of the three month follow up, the participant achieved three month abstinence. Otherwise, the self-reported number of cigarettes smoked per day was an indicator of treatment effectiveness. Participants’ nicotine dependence was evaluated using the Fagerstrom Test for Nicotine Dependence (FTND) score. Lifetime exposure to cigarettes was leveled by pack–year. Additional factors, such as the use of e-cigarettes, alcohol, betel nuts, and secondhand smoke exposure were also examined.

For secondary outcome assessment, we also assessed the quality of the SDM model using the SURE test (sure of myself, understand information, risk–benefit ratio, encouragement) for decisional conflict screening [28]. Adolescent smokers’ satisfaction toward the model was graded on a 5 point scale as follows: 5 = very good; 4 = good; 3 = neutral; 2 = bad; and 1 = very bad. The analysis was approved by the National Taiwan University Hospital Research Ethics Committee (201806018RIND).

## 3. Results

### 3.1. Participants

Table 1 shows that the participants had a mean age of 17.5 years and 95% were male and 5% were female. The number of years they had smoked ranged between 0 and 8, and 80% of participants had smoked for less than 5 years. The number of cigarettes participants smoked per day ranged between 2 and 40, and 45% and 25% of them smoked 10 (half a pack) and 20 (one pack) cigarettes per day, respectively. In total, 35% and 35% of participants had a history of less than one, or one to two pack–year, respectively. Pack–year was calculated as follows:

Number of cigarettes per day/20 (per pack) × Years smoked (Year)

In total, 45% of the participants reported using e-cigarettes or vapes, and 60% of them were exposed to secondhand smoke. A total of 50% of participants had an FTND score between 0 and 3, 20% of participants had an FTND score between 4 and 6, and 5% of participants had an FTND score between 7 and 10.

### 3.2. SDM Model Evaluation

#### 3.2.1. Important Factors Related to Adolescent Smoking Cessation

Table 2 shows the evaluation of SDM-model-related domains. Of all factors, the course completion difficulty, the time needed to complete course, and the effect on health brought about by cessation have a similar distribution of important factors related to adolescent smoking cessation. In total, 26% of participants considered treatment cost to be an important factor. The factors related to medication, such as smoking cessation rate, nicotine addiction level, and the adverse effect, were only rated as neutral among adolescent smokers.

#### 3.2.2. Replies to Comprehension Test about Treatments

A total of 47% of participants considered more counseling sessions to make cessation easier, whereas 37% were unsure about it. In total, 42% of participants considered all pharmacotherapies have an adverse effect and considered it better to not use them, and 47% of them were unsure about it. A total of 68% of participants considered professional psychological counseling to better handle the burden of emotional pressure. In total, 32% of participants considered cessation to be a matter of will, and that counseling would not help much, whereas 42% disagreed with this statement, and 26% were unsure about it. 

#### 3.2.3. SURE Test

In total, 100% of the participants gave positive feedback regarding the SURE test.

### 3.3. Satisfaction of the Cessation Model

As shown in Table 3, 50% of the participants were very satisfied with the sufficiency of the decision aids provided. A total of 43% of the participants considered the provided decision aids to be very helpful in increasing the cessation rate. In total, 57% of the participants were very satisfied with the content of the cessation model. A total of 57% of the participants were very satisfied with the location of the implementation of the cessation model. A total of 43% and 36% of the participants were very satisfied and satisfied with the cessation model, respectively.

### 3.4. Decreased Cigarettes Smoked per Day Participants and Point Abstinence Ratio

Table 4 shows the ratio of participants with a decreased number of cigarettes smoked per day and the point abstinence ratios of 1 week, 1 month, 3 months compared to baseline, respectively. In total, 60% of participants reported to have decreased the number of cigarettes smoked per day at 1 week and 1 month follow up; 70% of participants decreased the number of cigarettes smoked per day at the 3 month follow up compared to baseline. Totals of 15%, 20%, and 25% of participants reported abstinence of smoking at the 1 week, 1 month, and 3 month follow ups, respectively.

## 4. Discussion

### 4.1. Participants Characteristics

This study investigated 20 adolescents between 17 and 21 years of age, including 19 males and one female. Most of them had smoked for less than three years, smoking half to one pack of cigarettes per day; the pack–year varied, but was mostly under 3. These data indicate that our participants were mostly regular smokers without a long history of smoking and cigarette exposure; the time of initiation matches the age of junior high school to high school, where peer pressure becomes more influential [9]. Additionally, 60 percent of participants were exposed to secondhand smoke, which agrees with previous studies regarding environmental exposure influences. Regular smoking may be a result of either physiological or psychological addiction; using the distribution of the FTND score, we evaluated the dependence of nicotine and determined whether physiological or psychological addiction should be treated. An FTND score between 1 and 3 indicates low to mild nicotine dependence, a score between 4 and 6 shows moderate dependence, and a score between 7 and 10 represents high dependence. The distribution of FTND scores among participants remained even, between 1 and 6, which implies that psychological addiction is more dominant compared to physiological nicotine dependence. Therefore, cessation plans should actively shift from nicotine replacement therapy to a motivational interview and brief counseling among these adolescent smokers. 

### 4.2. SDM Model Evaluation

One essential component of SDM is to help patients understand their own preferences toward treatments. Among subjective important factors related to adolescent smoking cessation, a one-for-all factor of critically high importance was not found in the study. Under such circumstances, the SDM model is an ideal intervention for adolescent smoking cessation management to help adolescents make comparisons and decisions using a decision aid. For the cessation model, the treatment cost and time consumption seemed to have relatively higher importance for the participants. The cessation team and policy makers could focus on reducing both factors to increase cessation model usage. It is worth noting that cessation rate has a relatively low importance for adolescents, which may imply that the adolescents were still in the precontemplation stage without motivation before SDM intervention. These results reinforce the importance of motivational interviews and brief counseling in the “option talk” step.

The “help comparison” aspect in the SHARE approach and moving from the contemplation to action stage in the transtheoretical model both require the adolescent smokers to have a robust knowledge base. However, the results of comprehension tests in the “option talk” demonstrated a relatively high portion of uncertainty toward each question about cessation treatment, which may lead to a lack of confidence and motivation toward cessation treatments. Additionally, from the replies, we can conclude that there may be some misunderstanding of, and stereotypical beliefs toward, cessation treatment among adolescents. The pamphlet used in our model as a decision support tool was designed to have an “overview of the pros and cons of smoking cessation managements”, and the educators already helped the adolescents understand the pamphlet in their role of “coach” during “option talk”. The development of a decision aid conveying correct knowledge, encouraging acceptance, and that was more “attractive” for adolescent smokers is a future direction for improving the SDM model for adolescent smoking cessation. 

The SURE test (sure of myself, understand information, risk–benefit ratio, encouragement) was used to evaluate the quality of SDM and screen for decisional conflict. The SURE test showed a positive result for the quality of the complete SDM model, showing confidence, intention, and readiness of cessation, which are probable predictors of adolescent smoking cessation [29]. With this backdrop, the adolescent smokers were able to engage in preference-based informed decision making after entering the SDM model. The results showed that the model in the study can achieve the goal of SDM for adolescent smokers, which is also demonstrated in the satisfaction survey results toward the cessation model. 

By combining concepts of the three-talk model, transtheoretical model, and SHARE approach, our SDM model can take advantage of these widely adopted theory-based models. The transtheoretical model helps staging, and the SHARE approach with the three-talk model helps optimize the tailored decisions. The integration of “OARS” and “RULE” communication strategies into the SDM model can reinforce the decision process and quality. The SURE test can act as an indicator and confirmation of decision quality. The complete model can be further applied to different medical fields requiring customized decisions, especially in substance use disorders.

### 4.3. Effect on Smoking Cessation with SDM Integration

**Figure 2:**
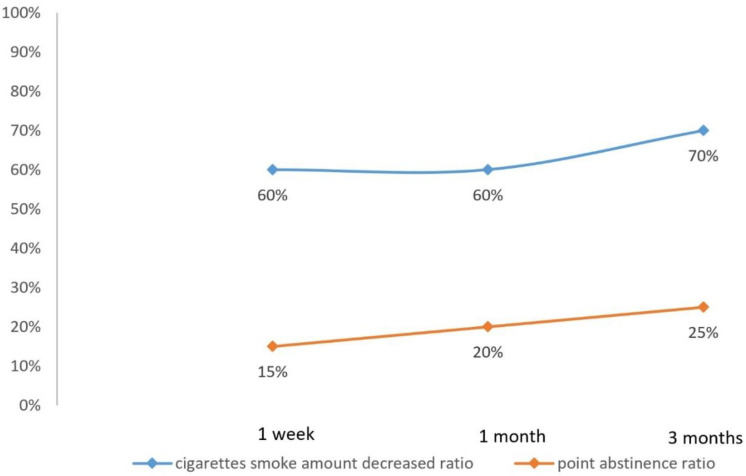
Cigarette smoke amount decreased ratio and point abstinence ratio.

As shown in Figure 2, the SDM-integrated cessation model had the effect of increasing 1 week, 1 month, and 3 month point abstinence ratios, achieving a positive primary outcome. Additionally, for participants who did not reach point abstinence, a positive result of a reduced daily number of cigarettes smoked was seen in 60 percent of participants at 1 week and 1 month follow up, and 70 percent at 3 month follow up. 

We can explain the increasing trend in the point abstinence ratio and cigarettes amount decrease ratio using the transtheoretical model, which was used in some smoking cessation models [30]. The integration of a decision aid, motivational interviews, and brief counseling into the “option talk” segment helped participants move into the action and maintenance stages. Barriers for the preparation and action stages were lowered by providing several smoking cessation options with high availability. Moreover, self-concept, self-reflection, and self- esteem were believed to be helpful and needed in adolescent smoking cessation [31]. The effective “option talk” brought about confidence and trust in adolescents and their decisions, making them more self-driven toward the maintenance stage after the “decision talk”. Additionally, regular follow up from 1 week, 1 month, and 3 months provided adolescents with stable maintenance and connection with the cessation team. If there is a relapse, the interactive SDM model can provide revisions regarding decisions and cessation plans.

Although the study only had a small number of participants, the comparison between the SDM-integrated model with previous cessation data in the US showed that the SDM-integrated model has a higher 3 month point abstinence rate (25%) than the individual counseling intervention (about 10%) and group counseling intervention (about 10%) [17]. 

### 4.4. Limitations

Several limitations should be noted in this study. First, because the attempt to use the SDM-integrated model is rather novel, there was difficulty in enrolling adolescents, which led to a limited sample size. The data may not be representative of all adolescent smokers due to possible selection bias. Second, the evaluation tools were originally designed for the control of tobacco use in adults [22]. The questionnaires used in the adolescent group may need further development and validation. Third, the length of follow up is also a limitation of the study. Our study used 3 month point abstinence as the primary outcome measurement. However, adolescent smoking is of high recurrence, and long-term follow up should be considered in future studies. Fourth, the effect and interaction between external factors influencing adolescent smoking, including families, peers, media, and social media should be further evaluated. Although the intervention of the SDM model had an indirect effect on these factors considering the effect of the “option talk”, the direct effect on these factors remains uncertain. Lastly, the limited sample size and lack of a control group made it difficult to quantify the association between the SDM model and abstinence rate and decreased cigarette number.

## 5. Conclusions

This cohort study revealed a positive effect on the adolescent point cessation rate and the potential benefits of the SDM-integrated model in the field of adolescent smoking cessation. The SDM concepts of three-talk and SHARE with the integration of motivational interviews and brief counseling may help adolescents to engage in informed preference-based decision making regarding smoking cessation. The SDM-integrated model can be applied to different fields of substance abuse disorders similar to tobacco in adolescents, reducing long-term potential health hazards. The dissemination of the model may assist more adolescent smokers in smoking cessation worldwide.

## Figures and Tables

**Table 1:** Demographic characteristics of participants.

Participants’ Characteristics	N (%) ^1^
Gender	
Male	19 (95)
Female	1(5)
Age	
16	3 (15)
17	6 (30)
18	6 (30)
19	4 (20)
21	1 (5)
Mean	17.5
Years smoked	
Less than one	2 (10)
1	4 (20)
1.5	1 (5)
2	5 (25)
3	4 (20)
5	2 (10)
6	1 (5)
8	1 (5)
Number of cigarettes per day	
2	1 (5)
4	2 (10)
5	1 (5)
7	1 (5)
10	9 (45)
20	5 (25)
40	1 (5)
Pack–Year	
Less than one	7 (35)
1–2	7 (35)
2–3	2 (10)
3–4	1 (5)
5–6	1 (5)
6–7	1 (5)
16–17	1 (5)
Betel nuts usage	
Yes	0 (0)
No	13 (65)
Ceased	2 (10)
Missing	5 (25)
E-cigarette or vape usage	
Yes	9 (45)
No	6 (30)
Missing	5 (25)
Alcohol usage	
Yes	5 (25)
No	10 (50)
Missing	5 (25)
Secondhand smoke exposure	
Yes	12 (60)
No	3 (15)
Missing	5 (25)
Education	
High school	20 (100)
FTND ^2^	
0	1 (5)
1	3 (15)
2	3 (15)
3	3 (15)
5	2 (10)
6	2 (10)
7	1 (5)
Unknown	5 (25)

^1^ Numbers and percentages unless otherwise stated. ^2^ FTND = Fagerstrom Test for Nicotine Dependence.

**Table 2:** SDM-model-related evaluation.

**Important factors related to adolescent smoking cessation**						
**Factor**	**Rating N (%) ^1^**					
	**0**	**1**	**2**	**3**	**4**	**5**
Nicotine addiction level	2 (11)	1 (5)	2 (11)	7 (37)	3 (16)	4 (21)
Cessation rate	4 (21)	1 (5)	5 (26)	5 (26)	1 (5)	3 (16)
Drug adverse effect	5 (26)	1 (5)	3 (16)	6 (32)	0 (0)	4 (21)
Treatment cost	4 (21)	2 (11)	1 (5)	3 (16)	4 (21)	5 (26)
Course completion difficulty	5 (26)	2 (11)	2 (11)	4 (21)	2 (11)	4 (21)
Time needed to complete course	5 (26)	1 (5)	2 (11)	4 (21)	3 (16)	4 (21)
Effect on health brought about by cessation	4 (21)	1 (5)	2 (11)	6 (32)	2 (11)	4 (21)
**Replies to comprehension test about treatments**						
**Question**	**Replies; N (%)**					
	**True**		**False**		**Not sure**	
1. The more times of counseling, the easier to achieve cessation.	9 (47)		3 (16)		7 (37)	
2. All pharmacotherapy have adverse effects, better not use them.	8 (42)		2 (11)		9 (47)	
3. Emotional pressure burden is better handled with professional psychological counseling.	13 (68)		1 (5)		5 (26)	
4. Smoking cessation is a matter of will, counseling won’t help much.	6 (32)		8 (42)		5 (26)	
**SURE test**						
**Factor**	**Participants’ replies; N (%)**					
	**Yes**			**No**		
1. Are you certain about your optimal choice?	19 (100)			0 (0)		
2. Are you clear about the risk and benefits of each choice?	19 (100)			0 (0)		
3. Are you certain about what risk and benefit has the most importance to you?	19 (100)			0 (0)		
4. Did you get enough help, opinions, and support to make decisions?	19 (100)			0 (0)		

^1^ From 0 to 5 indicates least important to most important.

**Table 3:** Satisfaction of cessation model.

Factors	Rating ^1^	N (%)
1. Do you consider the health educational decision aids provided by the cessation team sufficient?	5	7 (50)
	4	4 (29)
	3	3 (21)
	2	0 (0)
	1	0 (0)
2. Were decision aids provided by the cessation team helpful in increasing the confidence of successful cessation?	5	6 (43)
	4	5 (36)
	3	3 (21)
	2	0 (0)
	1	0 (0)
3. Are you satisfied with the content of the cessation model?	5	8 (57)
	4	3 (21)
	3	3 (21)
	2	0 (0)
	1	0 (0)
4. Are you satisfied with the location of implementing the cessation model?	5	8 (57)
	4	3 (21)
	3	3 (21)
	2	0 (0)
	1	0 (0)
5. Are you satisfied with the overall cessation model?	5	6 (43)
	4	5 (36)
	3	3 (21)
	2	0 (0)
	1	0 (0)

^1^ 5 = very good; 4 = good; 3 = neutral; 2 = bad; and 1 = very bad.

**Table 4:** Cigarette smoke amount decreased ratio and point abstinence ratio.

	1 Week	1 Month	3 Months
Decreased cigarettes smoked per day participants; N (%)	12 (60)	12 (60)	14 (70)
Point abstinence participants ratio; N (%)	3 (15)	4 (20)	5 (25)

## Data Availability

Data are available upon reasonable request.

## References

[1] Global Youth Tobacco Survey GYTS. https://www.hpa.gov.tw/Pages/Detail.aspx?nodeid=1725&pid=9931.

[2] World Health Organization (2018). WHO Global Report on Trends in Prevalence of Tobacco Smoking 2000–2025.

[3] Barrington-Trimis J.L., Braymiller J.L., Unger J.B., McConnell R., Stokes A., Leventhal A.M., Sargent J.D., Samet J.M., Goodwin R.D. (2020). Trends in the Age of Cigarette Smoking Initiation Among Young Adults in the US From 2002 to 2018. JAMA Netw. Open.

[4] Strong C., Juon H.-S., Ensminger M.E. (2016). Effect of Adolescent Cigarette Smoking on Adulthood Substance Use and Abuse: The Mediating Role of Educational Attainment. Subst. Use Misuse.

[5] Thacher J.D., Schultz E.S., Hallberg J., Hellberg U., Kull I., Thunqvist P., Pershagen G., Gustafsson P.M., Melén E., Bergström A. (2018). Tobacco smoke exposure in early life and adolescence in relation to lung function. Eur. Respir. J..

[6] Tam J., Brouwer A.F. (2021). Comparison of e-cigarette use prevalence and frequency by smoking status among youth in the United States, 2014–19. Addiction.

[7] Auf R., Trepka M.J., Selim M., Ben Taleb Z., De La Rosa M., Bastida E., Cano M.Á. (2019). E-cigarette use is associated with other tobacco use among US adolescents. Int. J. Public Health.

[8] Leonardi-Bee J., Jere M.L., Britton J. (2011). Exposure to parental and sibling smoking and the risk of smoking uptake in childhood and adolescence: A systematic review and meta-analysis. Thorax.

[9] Leshargie C.T., Alebel A., Kibret G.D., Birhanu M.Y., Mulugeta H., Malloy P., Wagnew F., Ewunetie A.A., Ketema D.B., Aderaw A. (2019). The impact of peer pressure on cigarette smoking among high school and university students in Ethiopia: A systemic review and meta-analysis. PLoS ONE.

[10] Lim K.H., Ghazali S.M., Lim H.L., Cheong K.C., Teh C.H., Lim K.K., Heng P.P., Cheah Y.K., Lim J.H. (2019). Smoking susceptibility among non-smoking school-going adolescents in Malaysia: Findings from a national school-based survey. BMJ Open.

[11] Pacek L.R., McClernon F.J., Bosworth H.B. (2018). Adherence to Pharmacological Smoking Cessation Interventions: A Literature Review and Synthesis of Correlates and Barriers. Nicotine Tob. Res..

[12] Zhang J., Kou G., Peng Y., Zhang Y. (2021). Estimating priorities from relative deviations in pairwise comparison matrices. Inf. Sci..

[13] Elwyn G., Laitner S., Coulter A., Walker E., Watson P., Thomson R. (2010). Implementing shared decision making in the NHS. BMJ.

[14] McClure J.B., Hartzler A.L., Catz S.L. (2016). Design Considerations for Smoking Cessation Apps: Feedback From Nicotine Dependence Treatment Providers and Smokers. JMIR Mhealth Uhealth.

[15] Willemsen M.C., Wiebing M., van Emst A., Zeeman G. (2006). Helping smokers to decide on the use of efficacious smoking cessation methods: A randomized controlled trial of a decision aid. Addiction.

[16] Rios L.E., Herval Á.M., Ferreira R.C., Freire M.d.C.M. (2019). Prevalences of Stages of Change for Smoking Cessation in Adolescents and Associated Factors: Systematic Review and Meta-Analysis. J. Adolesc. Health.

[17] Villanti A.C., West J.C., Klemperer E.M., Graham A.L., Mays D., Mermelstein R.J., Higgins S.T. (2020). Smoking-Cessation Interventions for U.S. Young Adults: Updated Systematic Review. Am. J. Prev. Med..

[18] Graham A.L., Amato M.S., Cha S., Jacobs M.A., Bottcher M.M., Papandonatos G.D. (2021). Effectiveness of a Vaping Cessation Text Message Program Among Young Adult e-Cigarette Users. JAMA Intern. Med..

[19] Chulasai P., Chinwong D., Chinwong S., Hall J.J., Vientong P. (2021). Feasibility of a Smoking Cessation Smartphone App (Quit with US) for Young Adult Smokers: A Single Arm, Pre-Post Study. Int. J. Environ. Res. Public Health.

[20] Elwyn G., Durand M.A., Song J., Aarts J., Barr P.J., Berger Z., Cochran N., Frosch D., Galasinski D., Gulbrandsen P. (2017). A three-talk model for shared decision making: Multistage consultation process. BMJ.

[21] Elwyn G., Frosch D., Thomson R., Joseph-Williams N., Lloyd A., Kinnersley P., Cording E., Tomson D., Dodd C., Rollnick S. (2012). Shared decision making: A model for clinical practice. J. Gen. Intern Med..

[22] Stiggelbout A.M., Pieterse A.H., De Haes J.C. (2015). Shared decision making: Concepts, evidence, and practice. Patient Educ. Couns..

[23] Rockville M. The SHARE Approach. https://www.ahrq.gov/health-literacy/professional-training/shared-decision/index.html.

[24] Joseph-Williams N., Newcombe R., Politi M., Durand M.A., Sivell S., Stacey D., O’Connor A., Volk R.J., Edwards A., Bennett C. (2014). Toward Minimum Standards for Certifying Patient Decision Aids: A Modified Delphi Consensus Process. Med. Decis. Mak..

[25] Lewis K.B., Wood B., Sepucha K.R., Thomson R.G., Stacey D. (2017). Quality of reporting of patient decision aids in recent randomized controlled trials: A descriptive synthesis and comparative analysis. Patient Educ. Couns..

[26] Miller W.R., Rose G.S. (2009). Toward a theory of motivational interviewing. Am. Psychol..

[27] Miller W.R., Rollnick S. (2009). Ten things that motivational interviewing is not. Behav. Cogn. Psychother..

[28] Legare F., Kearing S., Clay K., Gagnon S., D’Amours D., Rousseau M., O’Connor A. (2010). Are you SURE?: Assessing patient decisional conflict with a 4-item screening test. Can. Fam. Physician.

[29] Vallata A., O’Loughlin J., Cengelli S., Alla F. (2021). Predictors of Cigarette Smoking Cessation in Adolescents: A Systematic Review. J. Adolesc. Health.

[30] Aveyard P., Massey L., Parsons A., Manaseki S., Griffin C. (2009). The effect of Transtheoretical Model based interventions on smoking cessation. Soc. Sci. Med..

[31] Sim I., Hwang E., Sin B. (2020). A Self-Reflection Program for Smoking Cessation in Adolescents: A Phenomenological Study. Int. J. Environ. Res. Public Health.

